# How should we discuss genetic testing with women newly diagnosed with breast cancer? Design and implementation of a randomized controlled trial of two models of delivering education about treatment-focused genetic testing to younger women newly diagnosed with breast cancer

**DOI:** 10.1186/1471-2407-12-320

**Published:** 2012-07-28

**Authors:** Kaaren J Watts, Bettina Meiser, Gillian Mitchell, Judy Kirk, Christobel Saunders, Michelle Peate, Jessica Duffy, Patrick J Kelly, Margaret Gleeson, Kristine Barlow-Stewart, Belinda Rahman, Michael Friedlander, Kathy Tucker

**Affiliations:** 1 Department of Medical Oncology, Prince of Wales Hospital, High Street, Randwick, NSW, 2031, Australia; 2 Prince of Wales Clinical School, Faculty of Medicine, University of New South Wales, Kensington, NSW, 2052, Australia; 3 Peter MacCallum Cancer Centre, Familial Cancer Service, Locked Bag 1, A'Beckett Street, East Melbourne, VIC, 8006, Australia; 4 Familial Cancer Service, Westmead Hospital, Hawkesbury Road, Westmead, NSW, 2145, Australia; 5 Westmead Millennium Institute for Medical Research at the University of Sydney, PO Box 412, Westmead, NSW, 2145, Australia; 6 Department of Surgery, University of Western Australia, 35 Stirling Highway, Crawley, WA, 6009, Australia; 7 School of Public Health, The University of Sydney, Edward Ford Building, Sydney, NSW, 2006, Australia; 8 Hunter Family Cancer Service, PO Box 84, Waratah, NSW, 2298, Australia; 9 Centre for Genetics Education, PO Box 317, St Leonards, NSW, 1590, Australia

**Keywords:** Breast cancer, Genetic testing, *BRCA1*, *BRCA*2, Treatment, Clinical practice

## Abstract

**Background:**

Germline *BRCA1* and *BRCA2* mutation testing offered shortly after a breast cancer diagnosis to inform women’s treatment choices - treatment-focused genetic testing ‘TFGT’ - has entered clinical practice in specialist centers and is likely to be soon commonplace in acute breast cancer management, especially for younger women. Yet the optimal way to deliver information about TFGT to younger women newly diagnosed with breast cancer is not known, particularly for those who were not suspected of having a hereditary breast cancer syndrome prior to their cancer diagnosis. Also, little is known about the behavioral and psychosocial impact or cost effectiveness of educating patients about TFGT. This trial aims to examine the impact and efficiency of two models of educating younger women newly diagnosed with breast cancer about genetic testing in order to provide evidence for a safe and effective future clinical pathway for this service.

**Design/methods:**

In this non-inferiority randomized controlled trial, 140 women newly diagnosed with breast cancer (aged less than 50 years) are being recruited from nine cancer centers in Australia. Eligible women with either a significant family history of breast and/or ovarian cancer or with other high risk features suggestive of a mutation detection rate of > 10% are invited by their surgeon prior to mastectomy or radiotherapy. After completing the first questionnaire, participants are randomized to receive either: (a) an educational pamphlet about genetic testing (intervention) or (b) a genetic counseling appointment at a family cancer center (standard care). Each participant is offered genetic testing for germline *BRCA* mutations. Decision-related and psychosocial outcomes are assessed over 12 months and include decisional conflict (primary outcome);uptake of bilateral mastectomy and/or risk-reducing salpingo-oophorectomy; cancer-specific- and general distress; family involvement in decision making; and decision regret. A process-oriented retrospective online survey will examine health professionals’ attitudes toward TFGT; a health economic analysis will determine the cost effectiveness of the intervention.

**Discussion:**

This trial will provide crucial information about the impact, efficiency and cost effectiveness of an educational pamphlet designed to inform younger women newly diagnosed with breast cancer about genetic testing. Issues regarding implementation of the trial are discussed.

**Trial registration:**

The study is registered with the Australian and New Zealand Clinical Trials Group (Registration no: ACTRN12610000502033)

## Background

The clinical role of *BRCA1* and *BRCA2* mutation testing for younger women with breast cancer is in rapid transition because of advances in gene sequencing technologies and accumulating evidence for the contribution of *BRCA* mutation status to acute management of early breast cancer. Women diagnosed with breast cancer are offered genetic counseling and testing for germline mutations in *BRCA1* and *BRCA2* if they have a strong family history of the disease and/or they meet other criteria which point to a mutation detection rate that exceeds a predefined threshold for her local service. Genetic risk assessment has usually been offered on completion of surgery and adjuvant therapy for a new breast cancer, and routine genetic test results in Australia has to date taken between one and six months from blood draw. In contrast, genetic counseling and testing offered around the time of breast cancer diagnosis aims to provide the patient with genetic information that will assist in the choice of breast cancer treatment, primarily the choice between breast-conserving therapy (BCT) and mastectomy. The secondary effects of directing risk-reducing ovarian surgery and informing family members of their own cancer risks are not time dependent but useful outcomes of a genetic test at any time.

Women newly diagnosed with breast cancer with a *BRCA* mutation must choose whether to undergo BCT, unilateral mastectomy, or prophylactic bilateral mastectomies to prevent future breast cancers [1]. The incidence of another tumor developing in the treated breast increases in *BRCA* mutation carriers with longer follow-up [1] and it also varies with the type of local therapy [2]. Pierce et al. found that there was a significantly increased risk of local tumor recurrence in *BRCA1* and *BRCA2* mutation carriers treated with BCT compared to carriers treated with mastectomy at 10 years (10.5% versus 3.5%) and at 20 years (30.2% versus 5.5%) [2]. Compared with noncarriers, *BRCA1* and *BRCA2* mutation carriers have a substantially increased lifetime risk of contralateral breast cancer that is age dependent and can be up to 68%, if the age of the first cancer is <40 [3,4]. While there is no evidence that prophylactic mastectomy improves breast cancer survival for *BRCA* mutation carriers [5], the risk of and potential emotional impact of a subsequent breast cancer and the need for further treatment are important issues to consider [1]. Contralateral prophylactic mastectomy can decrease the risk of subsequent breast cancer by up to 95% [6-8]. The *BRCA* mutations also confer a 13-46% lifetime risk of ovarian cancer [9]. The secondary breast cancer prevention role of premenopausal risk-reducing bilateral salpingo-oophorectomy (RRSO) is less well established. Although there is an important breast cancer risk reduction of between 39% (*BRCA1*) and 72% (*BRCA2*) [10] among mutation carriers who have RRSO before the age of 50 years, Domchek et al. 2010 did not find a similar reduction in women who had had prior breast cancer [8]. *BRCA1* and *BRCA2* mutation testing can now be made available rapidly, within 5 working days if required. Soon, further advances in sequencing will substantially reduce the cost of genetic analysis [11] and will open the opportunity for genetic testing to even more women who might benefit from this information. This means that a woman’s *BRCA* mutation status can feasibly be used now to inform her surgical decisions regarding BCT or mastectomy (unilateral or bilateral). In the future, timely *BRCA* mutation testing will extend to selection of specific adjuvant systemic chemotherapy once the optimal systemic therapy for *BRCA* mutation carriers is established, including the role of poly (ADP-ribose) polymerase (PARP) inhibitors [12,13] and platinum-based chemotherapy. Hereafter, genetic counseling and testing offered shortly after a women’s diagnosis of breast cancer will be referred to as ‘treatment-focused genetic testing’ (TFGT). The potential for ever increasing scope for the use of TFGT in acute breast cancer management in the very near future means that new models for genetic counseling will be needed in order to manage the increased demand for and delivery of genetic information. In particular, there is a pressing need to develop cost effective clinical pathways which utilize the multidisciplinary cancer and genetics team, in order to offer TFGT in a streamlined way which is acceptable to patients and health care providers.

A concern shared by both patients and health professionals is that TFGT may create undue psychological burden among women diagnosed with breast cancer at a very vulnerable time in their life [14]. There is limited empirical data available on the psychosocial implications of TFGT. Two studies have described the behavioral and psychological impact of TFGT in the US [15-17] and The Netherlands [18]. In the US study, 194 patients newly diagnosed with breast cancer were offered genetic testing before definitive treatment and the impact on surgical decision-making was evaluated. Definitive treatment was defined as mastectomy (unilateral or bilateral) or BCT, including commencement of radiation treatment [15]. Forty-eight percent of women who were found to carry a *BRCA1* or *BRCA2* mutation opted for bilateral mastectomy (BM), compared to 4% of women who declined genetic testing. Compared to women who chose BCT or unilateral mastectomy, those who chose BM did not report diminished quality of life or increased distress [17]. The Dutch prospective study assessed the psychological impact of TFGT in women diagnosed with breast cancer who were about to commence adjuvant radiotherapy. Patients’ distress levels did not increase after genetic counseling and testing [18]. Another randomized controlled trial is currently in progress in The Netherlands, which is assessing the impact of rapid genetic testing and counseling on women newly diagnosed with breast cancer on surgical decision making and psychosocial outcomes [19].

A limitation of these studies is that they have selected or are selecting women based primarily on a significant family history of breast and/or ovarian cancer [16,18] or have retrospectively selected women based upon their *BRCA* mutation status [14]. In addition, these previous studies have not included a health economic analysis assessing the cost effectiveness of delivering TFGT to women diagnosed with breast cancer. The concept of a strong family history as the optimum criterion for selection for genetic testing is being challenged with evidence for several personal and disease characteristics that are suggestive of *BRCA* mutations. For example, between 30-50% of women with a *BRCA1/2* mutation have no significant or known family history of breast or ovarian cancer [20,21] and up to 8% of women with Ashkenazi Jewish ancestry and breast cancer diagnosed under 50 years have no relevant family history [22]. Up to 27% of women under the age of 50 and 36% of women diagnosed at or under age 40, unselected for family history with triple negative breast cancer are *BRCA1* mutation carriers [23]. Of over 12,000 new breast cancer diagnoses in Australia annually, 24% are in women under 50 [24]. In relation to genetic testing, the predictive value of having a *BRCA* mutation in these younger age-of-onset cases increases especially when combined with triple negative disease [25]. Yet little is known about the acceptability of TFGT among younger women without a relevant cancer family history but with disease, tumor or ethnic features suggestive of a high risk of actually having a *BRCA* mutation. Meiser and colleagues conducted in-depth semi-structured interviews with 26 younger women (aged 50 years or less) diagnosed with breast cancer (14 had undergone TFGT and 12 had not), which explored their actual and hypothetical attitudes toward, and experiences (if any) of TFGT [26], and their information preferences regarding TFGT [27]. Women with and without a relevant family history of breast and/or ovarian cancer were included. All of the participants viewed TFGT as highly acceptable and wanted to receive information about it early, either at diagnosis or shortly thereafter, to inform their treatment options and to assist family members. Women preferred to receive the offer of TFGT verbally in a face-to-face consultation with a health professional. However, they also highlighted the importance of provision of supportive brief written information about TFGT that they could take away and consider at home [27].

In this paper, we report the design of a study that compares two different models of delivering information about TFGT to younger women newly diagnosed with breast cancer. We also report several key issues encountered in the implementation of the study, which will have important implications for the integration of TFGT into acute breast cancer management in the near future.

### Study objectives and hypotheses

**Figure 1:**
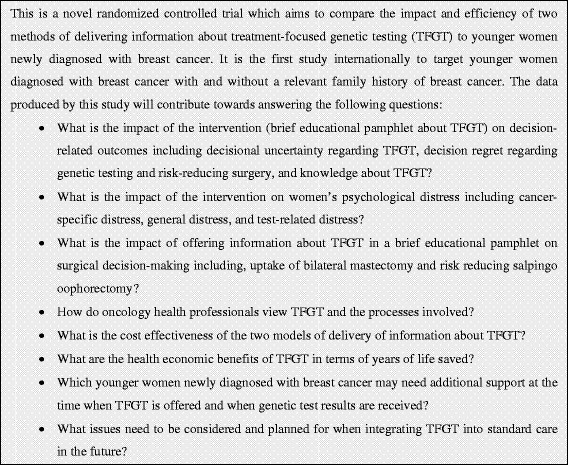
Research questions addressed in the study.

The aim of this study is to compare the behavioral and psychosocial impact, efficiency and safety of offering information about TFGT to younger women newly diagnosed with breast cancer (a) using a brief educational pamphlet about TFGT (educational materials, ‘EM’ intervention) or (b) using pre-test genetic counseling at a family cancer service (standard care, ‘SC’ control). The efficiency of offering information about TFGT will be operationalised in a health economic analysis and through an assessment of health professionals’ views on the effectiveness of the TFGT process. The safety of the intervention will be determined by assessing its impact on psychological distress. This is a non-inferiority trial, in which our primary hypothesis is that patient decisional conflict regarding TFGT will be no worse in the intervention compared to that of the control group. A summary of the research questions that are addressed in the study are presented in Figure 1.

Our secondary hypotheses are: (i) that other decision-related and psychological outcomes (including decision regret regarding TFGT and surgery; anxiety; depression; cancer-specific distress; test-related experiences and distress; knowledge of TFGT; and uptake of genetic testing) will not be inferior in the intervention group compared to the control group, and (ii) that women who opt for TFGT will have a higher uptake of BM compared to data from the Royal Australasian College of Surgeons National Breast Cancer Audit on over 12,000 women with early breast cancer diagnosed annually [28].

### Design

**Figure 2:**
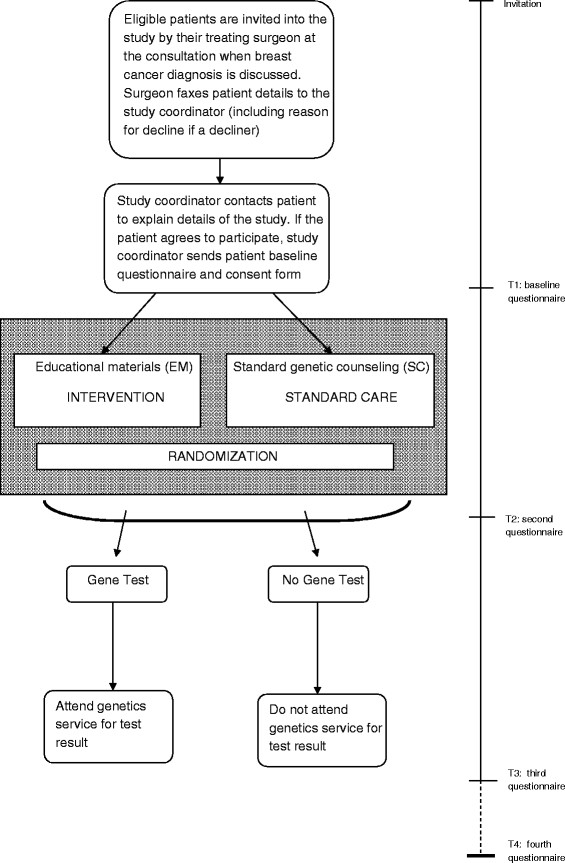
Study design and recruitment flow.

This is a multicenter randomized controlled trial which is being conducted at nine hospital sites located in three states in Australia (New South Wales, Victoria and Queensland) in two stages. The study protocol adheres to CONSORT guidelines [29]. The study design is presented in Figure 2. In Stage I, 140 eligible patients are being randomized to receive either EM or SC. Randomization is in a 1:1 ratio and is achieved through a computerized random sequence, which is generated by an independent person. Each number in the sequence is printed and placed in an unmarked envelope such that allocation is concealed from the study coordinator until randomization occurs. It is not possible to blind participants to their allocation because they become aware of it once the genetics staff arrange patients’ genetic counseling appointments. The primary outcome measurement is level of decisional conflict regarding TFGT. The secondary behavioral and psychological outcome measures include: (i) uptake of TFGT; (ii) uptake of BM; (iii) cancer-specific distress; (iv) general anxiety and depression; (v) distress associated with decision to have genetic testing; (vi) level of decision regret regarding TFGT and surgical decisions, and (vii) family involvement in decision-making. Over the course of the study, four questionnaires will be administered: at baseline (prior to randomization); one week after receiving education about TFGT; 3 weeks after receipt of genetic testing result (or at the equivalent time point for those who do not have genetic testing or who choose not to receive their test result); and 12 months after study enrolment. 

A health economic analysis is also included with the objective of ascertaining the cost effectiveness of the intervention compared to standard care, and the potential benefit of the intervention in terms of number of life years saved. The health economic analysis is modeled on similar evaluations conducted in a cancer/medical setting [30]. The costs associated with each mode of information delivery are collected from each site by the study coordinator, including printing costs, blood collection fees, and courier fees. Surgeons’ and genetic counselors’ time (minutes) associated with discussing TFGT with each patient is recorded by each practitioner on a record sheet, which is faxed to the study coordinator after the relevant appointment. In Stage II, a retrospective health professionals’ survey will be conducted to determine health care providers’ attitudes toward and experiences of the TFGT process. The survey is targeted to breast surgeons, medical oncologists, radiation oncologists, and breast care nurses involved in the delivery of TFGT. Ethics approval has been received from the institutional review board for each site.

## Methods

### Participants

**Figure 3:**
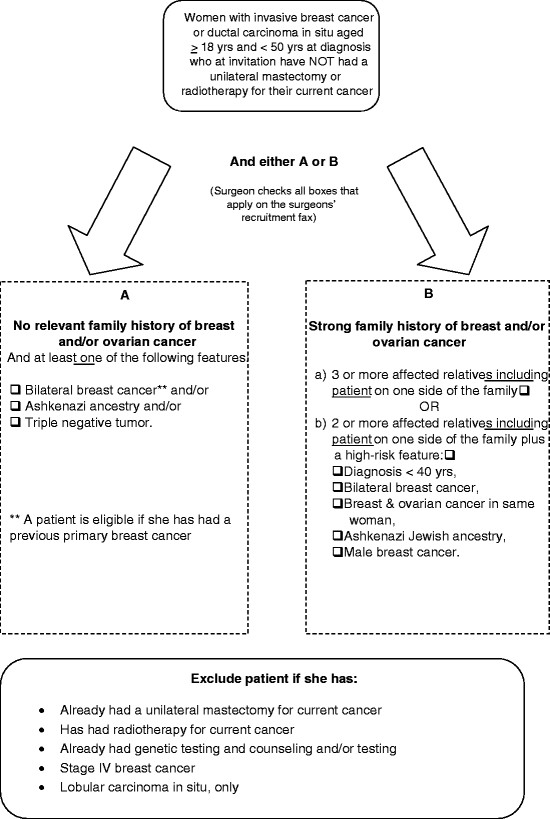
Study inclusion and exclusion criteria.

A woman is eligible to participate if she is aged less than 50 years at diagnosis of invasive breast cancer or ductal carcinoma *in situ* and has not had a mastectomy for her current cancer or radiotherapy at the time of invitation. The eligibility and exclusion criteria are presented in detail in Figure 3. The patient must have (A) either a strong family history of breast and/or ovarian cancer, specifically (i) three or more affected relatives including the patient on one side of the family or (ii) two or more affected relatives including the patient on one side of the family and a high-risk feature [31], or (B) in the absence of a relevant family history, the woman must have at least one of the following high risk features: bilateral breast cancer; and/or Ashkenazi Jewish heritage, and/or a triple negative tumor. 

### Intervention

The educational materials entitled ‘Making a decision about treatment-focused genetic testing’ were developed by the research team on the basis of the information preferences expressed by women in our qualitative interviews. The development and pilot-testing of the educational materials is reported in detail elsewhere [27]. The brief educational pamphlet contains information about: (i) what TFGT is; (ii) the purpose of TFGT; (iii) why a woman might consider having TFGT; (iv) what is involved in having TFGT; and (v) different outcomes of TFGT and their implications for the patient and her family.

Participants in the intervention group who elect to have TFGT receive an intake interview over the telephone, when their family history is collected, including cancer history and ages of diagnoses of the patient’s biological first- and second-degree relatives, approximately one week prior to their results disclosure appointment.

### Control

Participants allocated to the control group receive pre-test genetic counseling ranging from 40-60 minutes in duration, which typically covers the following topics:

(i) Explanation of hereditary breast cancer and the breast cancer ‘protection genes’ (*BRCA1*/*BRCA2*);

(ii) Discussion of patient’s risk of having a *BRCA* mutation based upon their family history or other high-risk features;

(iii) What a *BRCA1/BRCA2* mutation search involves;

(iv) Explanation of the potential advantages and disadvantages of genetic testing according to the patient’s family history and personal situation;

(v) An explanation of what type of information genetic testing may or may not provide and the implications for treatment/prevention;

(vi) Checking patient understanding of the concepts discussed;

(vii) Obtaining informed consent for genetic testing if the patient wishes to proceed.

Participants in the control group are contacted by telephone by the genetic counselor prior to their pre-test appointment for a brief intake interview equivalent to the intervention group.

### Genetic testing and disclosure of test results

Participants are offered TFGT with a turn around time of 10 working days from blood draw, free of charge, as part of the study.

All participants who elect to have TFGT receive their test results in a face-to-face appointment at a family cancer service attached to their treating hospital, regardless of their randomization. The results disclosure appointment of 20-40 minutes typically covers the following content:

(i) Disclosure and explanation of the test result;

(ii) Implications of the result for the patient’s personal risk of another primary breast cancer and risk of ovarian cancer;

(iii) Options for reduction of future risk of these cancers;

(iv) Implications of the test result for genetic relatives;

(v) Communicating test results with genetic relatives, informed consent and privacy issues;

(vi) Checking patient understanding of the concepts discussed.

### Recruitment

Recruitment commenced in July 2010 and details of the process are presented in Figure 2. The study coordinator sends the first questionnaire and a consent form to each patient who agrees to participate, either online or by post. The patient is randomized immediately after the study coordinator receives her completed questionnaire and consent form.

### Measures

#### Patient questionnaires

**Figure 4:**
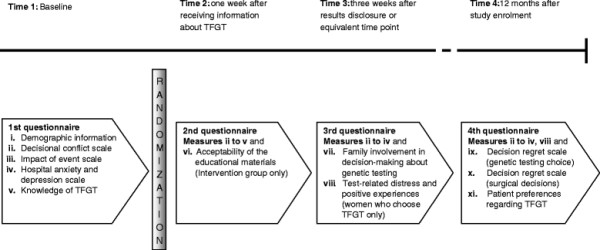
Patient measures administered during the study.

The four self-administered patient questionnaires are completed over a 12 month period. A summary of the measures included at each time point is provided in Figure 4. Details of the measures are provided below:

(i) *Demographic, disease- and family history-related data:* Sociodemographic data are collected including age, marital status, language spoken at home, education, employment, occupation, and parity.

(ii) *Decisional conflict scale (DCS):* This 10-item validated scale measures decisional conflict in relation to genetic testing choices, including uncertainty about alternatives, modifiable factors contributing to uncertainty, and perceptions of effectiveness of decision-making [32]. Response options are 0 = ‘yes’ 2 = ‘unsure’ and 4 = ‘no’. Scores are converted to a 0-100 scale by summing scores, dividing by 10 and then multiplying by 25, with higher scores indicating greater decisional conflict [33].

(iii) *Impact of Event Scale (IES):* The 15-item IES, which has been validated in this population [34], is being used to measure the frequency and severity of breast cancer specific worry. Response options are 0 - ‘not at all’, 1 = ‘rarely’, 3 = ‘sometimes’, and 5 = ‘often’. A total score is obtained by summing the items (range 0 to 75) with a higher score indicating more distress.

(iv) *Hospital Anxiety and Depression Scale (HADS):* The 14-item HADS is a widely used measure of emotional disturbance and has two subscales measuring anxiety and depression [35]. Each question has four possible responses. Responses are scored on a scale from 0 to 3. A total scale score is obtained by summing each item (range 0 = 42) with a higher score indicating more general distress.

(v) *Knowledge of TFGT:* Ten items were purpose designed and pilot-tested for this study to assess knowledge and understanding of TFGT. There are three response options including ‘true’, ‘false’ and ‘don’t know’. Correct responses are assigned a score of ‘1’ and incorrect or ‘don’t know’ responses are scored ‘0’. Correct answers are summed to give a score out of 10 (range: 0-10).

(vi) *Acceptability of the educational materials* (intervention group only): have The length, pace, amount of information, and balance and whether other family members used the educational materials is assessed using five structured response categories [36].

(vii) *Family involvement in decision-making about genetic testing:* Two items assess whether other family members participated in the decision-making process.

(viii) *Test-Related Distress and Positive Experiences* (women who choose testing only): This measure includes 10 items from a validated questionnaire (the Multidimensional Impact of Risk Assessment Scale) [37] assessing distress (six items) and positive experiences (four items) about genetic testing. Response options range from 0 = ‘never’ to 5 = ‘often’ with scores ranging from 0 to 30 and 0 to 20 for the distress and positive experiences scales, respectively.

(ix) *Decision Regret Scale (DRS: Genetic Testing Choice*): This five-item scale measures distress or remorse after health care decisions, and has excellent psychometric properties [38]. It has been adapted to measure decision regret in relation to women’s decision regarding genetic testing. Response options range from 1 = ‘strongly agree’ to 5 = ‘strongly disagree’. Two items are reverse scored. Scores are converted to a 0-100 scale by subtracting 1 from each item and then multiplying by 25. To obtain a final score, each item is summed and averaged with higher scores indicating more regret (0 = no regret and 100 = high regret) [39].

(x) *Decision Regret Scale (DRS: Surgical Decisions):* A second version of the DRS measures decision regret in relation to women’s decisions about bilateral mastectomy and RRSO.

(xi) *Patient preferences regarding TFGT:* Two items have been adapted from a previous study conducted by our team exploring patient preferences and perceived survival benefits among younger women with early breast cancer considering endocrine therapy [40]. The items assess: (i) the minimum perceived mutation carrier rate required to make TFGT worthwhile; and (ii) how much women would be willing to pay for such a test.

Clinical information will be gathered from participants’ medical records retrospectively including family history of breast and/or ovarian cancer; date of diagnosis; tumor size and grade; nodal and receptor status; menopausal status at diagnosis; type and date of surgery (lumpectomy, unilateral mastectomy and BM, axillary surgery, reconstruction surgery, RRSO–including intended RRSO); adjuvant treatment/s, and *BRCA* mutation status.

#### Health professionals’ questionnaire

The 17 item online retrospective survey has been developed for the current study using items adapted from a previous health service evaluation [41] and also based upon expert input from the research team. The survey will be administered once all participants have been recruited to the study. Oncology health professionals who have participated in the current trial or who were directly involved in the care of women involved in the trial, will be invited to participate via email with a secure link provided therein to the consent form and online self-report questionnaire. The survey aims to assess these health professionals’ attitudes and experiences of the TFGT trial, including their satisfaction with the trial and the TFGT process; perceived success of the trial; the amount of health professionals' time invested in providing the TFGT service; perceived barriers and enablers to delivering TFGT effectively; aspects of the TFGT process that could be improved; and suggestions for integrating TFGT into clinical practice.

### Statistical power

It is estimated that less than 10% of breast cancer patients of all ages will be eligible for the study.

A total sample size of 128 has 80% power to test the non-inferiority of the intervention on the primary outcome, the Decisional Conflict Scale (DCS). For the DCS, a non-inferiority margin will be -10 units, that is, the intervention group will be considered no worse than the standard care group if the 95% CI of the mean difference in DCS between the two groups lies wholly above -10 units. The DCS has 10 items, with the following response options: ‘Yes’, ‘Unsure’ and ‘No’ to items such as: ‘Do you know which options are available to you?’ The DCS total score ranges from 0 to 100 and thus a non-inferiority margin of -10 units on the DCS corresponds to only one of the 10 items having been endorsed as ‘No’ instead of ‘Yes’. The power calculation is based on a 5% significance level (2-sided), a true mean difference of zero units and a conservative SD of 20 units.

### Statistical analysis

An intention-to-treat analysis will be conducted on the differences in means between the intervention and standard care groups on psychological and decision-related outcomes. Linear regression shall be used for each outcome measured at 12 months, adjusting for the patient baseline scores. Further multivariate analyses will be used if preliminary analyses suggest a need to adjust for potential confounding variables (e.g. sociodemographic characteristics including age, parity, education, mutation status, and family history). In order to investigate whether, and how, patient outcomes differ between the intervention and standard care groups over time, the repeated measurements (T1, T2, T3, T4) shall be analyzed using linear mixed effects [42]. This approach will appropriately adjust for the repeated measures per person and allows for missing values. For all analyses, appropriate model checking will be conducted and where required, alternatives to linear regression will be used (e.g., logistic regression, if there is a need to dichotomise an outcome variable).

## Discussion

This study aims to compare the impact, efficiency and safety of two different ways of offering information about TFGT to women using (a) brief educational materials or (b) standard care (pre-test genetic counseling). In June 2012, 110 participants have been recruited to the study. During the recruitment period, several issues have been encountered that warrant consideration to ensure successful integration of TFGT into standard clinical practice for women diagnosed with breast cancer in the future.

### A multidisciplinary approach

The treating surgeon is responsible for inviting eligible patients into the study and for completing the recruitment fax (analogous to a referral letter). Therefore, the surgeon has the role of ‘gatekeeper’ of delivery of brief initial information about TFGT, which is likely to be the case in the future clinical setting. Some eligible patients, however may be missed by the surgeon if he or she does not recognize the patient as being eligible, forgets to invite them or does not have time to complete the recruitment sheet. Hence, a multidisciplinary and streamlined approach is required to ensure that all patients who are eligible for testing are informed about the opportunity to have TFGT. New models of ascertainment will need to be developed using the members of the multidisciplinary team.

### Results disclosure

The hospital sites in the current study are located in major cities, or in large regional centers, so patients referred for recruitment to the study have access to a dedicated onsite family cancer service, which is staffed by at least one genetic counselor and a genetics specialist. Genetic test results are disclosed to each patient who elects to have TFGT in a face-to-face appointment with the genetics team. However, if TFGT is to be expanded across clinical services, rapid access to a genetics practitioner is unlikely to be possible especially in smaller rural and regional hospitals, where visiting non-genetics specialists may need to deliver the results of genetic testing to the patient. One alternative that is already utilized by cancer genetics practitioners for oncology patients located in rural or remote areas is disclosure of genetic test results via telehealth technology (‘telegenetics’), with the genetics specialist providing the consultation from their city or regional hospital site. This model may be feasible for the TFGT context given a recent study conducted with 195 Australian women with a moderate or high-risk family history of hereditary breast and ovarian cancer seeking genetic counseling, in which telegenetics was found to be equally effective to face-to-face genetic counseling [43].

### Process-related issues

The process-related issues that have emerged in offering TFGT at our nine hospital sites have important implications for effective integration of TFGT into clinical practice. The delivery of the genetic testing result to the genetics specialist and/or genetic counselor by the test laboratory staff in a password-protected email is efficient and confidential. Blood collection for patients in the intervention arm, either at a local pathology service or by a general physician, has on several occasions been problematic, with some delays experienced in pick up of the blood by the courier for transport to the test laboratory. When TFGT is integrated into standard clinical pathways for women with breast cancer, blood will be collected according to usual hospital protocols avoiding this issue. For patients in rural or remote areas, establishing time efficient protocols for sample processing will be an important part of the service set up. If family history is to be used as a selection factor, timely verification of the reported cancer diagnoses will need to be considered. While individuals can report family histories of cancer accurately [44], it is possible that some patients will not meet the criteria for genetic testing after their family history is verified. One way of addressing inaccurate or incomplete collection of family history by the referring clinician is for him or her to direct each patient referred for TFGT to a website with a family history questionnaire, which the patient can complete. Online completion of a brief pre-test family history checklist that is accessible directly by genetics staff will be an important way of streamlining collection of this data, in preparation for more widespread use of TFGT in the near future.

### TFGT is too much too soon for some women

To date, although 100% of 110 women who have enrolled in the study have opted to have TFGT, a small proportion of women (8.2%) invited to participate have either declined participation when invited by the surgeon (*n* = 4, 3.6%) or after initial contact with the study co-ordinator (*n* = 5, 4.6%). Several women reported that their reason for declining was that they felt overwhelmed and could not cope with additional decision-making when dealing with a breast cancer diagnosis. This is in keeping with the findings of focus groups conducted with 13 women diagnosed with breast cancer who were identified *BRCA* mutation carriers [14]. The women were asked to provide their hypothetical views on TFGT. The majority of women reported that they would find it too overwhelming at the time of diagnosis to consider genetic testing. In the clinical setting, it will be important to set up formal processes to ensure that women who meet criteria for TFGT, but who do not want to consider genetic testing at the time of their diagnosis, are given the option of referral to a family cancer service at a later date after their definitive breast cancer treatment has finished and they are ready to consider genetic issues.

### Methodological strengths and limitations

The study is a randomized controlled non-inferiority trial, which is a robust and scientifically rigorous design for addressing the research questions. Wherever possible, the study employs validated measures that have been utilized previously with women diagnosed with breast cancer. A substantive strength of the study is that outcomes for both patients and oncology health professionals are being assessed. A comprehensive health economic analysis of the two models of information delivery is also included. Together, these data will contribute to effective planning for the integration of TFGT into standard care for women diagnosed with breast cancer in the future. The multicenter nature of the study will also increase the external validity and generalization of the study findings. The sample size in the current study using individual randomization will provide sufficient power to detect clinically meaningful effects for the key outcome variable of decisional conflict. Two potential limitations of the study must also be acknowledged. It was beyond the scope of the research to translate the patient questionnaires into other languages. Hence, women from non-English speaking backgrounds could not be included. In order to avoid contamination, we were also unable to include women who had previously attended a genetics service when unaffected by breast cancer, who may have benefited from TFGT.

## Conclusion

This randomized controlled trial will determine the behavioral and psychosocial impact and efficiency of two models of delivering information about genetic testing to younger women newly diagnosed with breast cancer. The trial will answer important questions about whether brief patient educational materials about TFGT are a safe and effective method of assisting women to make an informed decision about genetic testing at a stressful time. The evaluation of both patient outcomes and health professionals’ views regarding the TFGT process, together with the health economic analysis, will facilitate successful planning for the integration of TFGT into routine acute breast cancer management.

## Competing interests

The authors declare that they have no competing interests.

## Authors’ contributions

BM, GM, JK and KT conceived of the study. KW, BM, GM, JK, CS, MP, JD, PK, MG, KBS, BR, MF, and KT for the TFGT Collaborative Group, participated in the design and coordination of the study. KW wrote the first draft of the manuscript and all co-authors have been involved in reviewing drafts of the manuscript and revising it critically for important intellectual content. BR has a lead role in coordination of the study. All authors named in the TFGT Collaborative Group have made substantial contributions to the acquisition of data. All authors have provided their final approval of the current version of the manuscript to be published.

## TFGT Collaborative Group

The additional members of the Treatment Focused Genetic Testing Collaborative Group are in alphabetical order of group or institution: Cabrini Private Hospital, Melbourne (Y. Antill, P. Gregory, L. Lipton, L. McKay, J. Senior); Calvary Health Care Sydney and Cunningham Centre for Palliative Care, Sydney (E.A. Lobb); Department of Medical Oncology, Prince of Wales Hospital (P. Crowe, A. Matthews, G. Neil, A. Parasyn, D. Thomson, E. Zilliacus); Hereditary Cancer Clinic, Prince of Wales Hospital, Sydney (L. Andrews, J. Gale); Monash Medical Centre, Melbourne (J. Fox, M. Harris, S. Hart, C. Smythe, M. White); Nambour Hospital, Nambour (L. Creighton, K. Crowe, J. D’arcy, S. Grieve, E. Secomb); Peter MacCallum Cancer Centre, Melbourne (L. Cicciarelli, M. Henderson, J. O’Brien, C. Poliness); Royal Brisbane and Women's Hospital (A. Hattam, R. Susman, O. Ung,); Royal North Shore Hospital, Sydney (R. Dickson, M. Field, K. Moore); St George Hospital, Sydney (P. Bastick, S. Inder, J. Lynch, P. Schwartz, R. Zia); The Poche Centre, Sydney (C. Mak, K. Snook, A. Spillane); University of Melbourne (J. Hopper); University of Western Australia, Perth (L. Geelhoed); Westmead Hospital, Sydney (M. Bowman, D. Cheung, S. Edirimanne, E. Edwards, E. Elder, J. French, D. Moon).

## Pre-publication history

The pre-publication history for this paper can be accessed here:

http://www.biomedcentral.com/1471-2407/12/320/prepub
